# Three new species of *Columnea* (Gesneriaceae) from the western Andean slopes of Ecuador and Colombia

**DOI:** 10.3897/phytokeys.182.69016

**Published:** 2021-09-20

**Authors:** John L. Clark, Francisco Tobar, Laura Clavijo, Mathieu Perret, Catherine Helen Graham

**Affiliations:** 1 Science Department, The Lawrenceville School, Lawrenceville, NJ 08648, USA The Lawrenceville School Lawrenceville United States of America; 2 Área de Investigación y Monitoreo de Avifauna, Aves y Conservación – BirdLife en Ecuador, Quito, Ecuador Área de Investigación y Monitoreo de Avifauna, Aves y Conservación – BirdLife en Ecuador Quito Ecuador; 3 Instituto de Ciencias Naturales, Universidad Nacional de Colombia, Bogotá, Colombia Instituto Nacional de Biodiversidad, Herbario Nacional del Ecuador Quito Ecuador; 4 Instituto Nacional de Biodiversidad, Herbario Nacional del Ecuador QCNE, Quito, Ecuador Universidad Nacional de Colombia Bogotá Colombia; 5 Conservatoire et Jardin botaniques de la Ville de Genève, Ch. de l’Impératrice 1, CH-1292 Chambésy, Switzerland Conservatoire et Jardin botaniques de la Ville de Genève Chambésy Switzerland; 6 Biodiversity and Conservation Biology Unit, Swiss Federal Research Institute WSL, Birmensdorf, Switzerland Swiss Federal Research Institute Birmensdorf Switzerland

**Keywords:** Colombia, *
Columnea
*, Ecuador, Gesneriaceae, taxonomy

## Abstract

Three new species of *Columnea* (Gesneriaceae, tribe Gesnerieae) are described from the western Andean slopes of northern Ecuador and southern Colombia. *Columneaangulata* J.L. Clark & F. Tobar and *Columneafloribunda* F. Tobar & J.L. Clark are described from northern Ecuador. *Columneatecta* J.L. Clark & Clavijo is described from southern Colombia and northern Ecuador. The three new species are facultative epiphytes with dorsiventral shoots and are readily recognized by bright red tips on the abaxial and adaxial leaf surfaces. The species described here are vegetatively similar to the sympatric species *Columneapicta* H. Karst. and are readily differentiated by floral features that are illustrated, described and featured with digital images.

## Abstract (es)

Se describen tres especies nuevas de *Columnea* (Gesneriaceae, tribu Gesnerieae) originarias de la vertiente occidental de los Andes de Ecuador y Colombia. *Columneaangulata* J.L. Clark & F. Tobar y *Columneafloribunda* F. Tobar & J.L. Clark se describen del norte de Ecuador; *Columneatecta* J.L. Clark & Clavijo se describe del sur de Colombia y norte de Ecuador. Las tres especies nuevas son epífitas facultativas con vástagos dorsiventrales, las cuales se reconocen fácilmente por las puntas de las hojas de color rojo brillante en ambas superficies; son vegetativamente similares y simpátricas con *Columneapicta* H. Karst., pero se pueden diferenciar por las características florales que se ilustran, describen y detallan con imágenes digitales en este artículo.

## Introduction

The flowering plant family Gesneriaceae, with over 3400 species and 150+ genera (Weber 2004; Weber et al. 2013), is in the order Lamiales. The family is divided into three subfamilies and seven tribes (Weber et al. 2013, 2020), which represent monophyletic lineages (Ogutcen et al. 2021). The majority of New World members are in the subfamily Gesnerioideae and are represented by 1200+ species and 77 genera (Clark et al. 2020). *Columnea* L. is classified in the tribe Gesnerieae and subtribe Columneinae (Weber et al. 2013, 2020).

**Figure 1.**
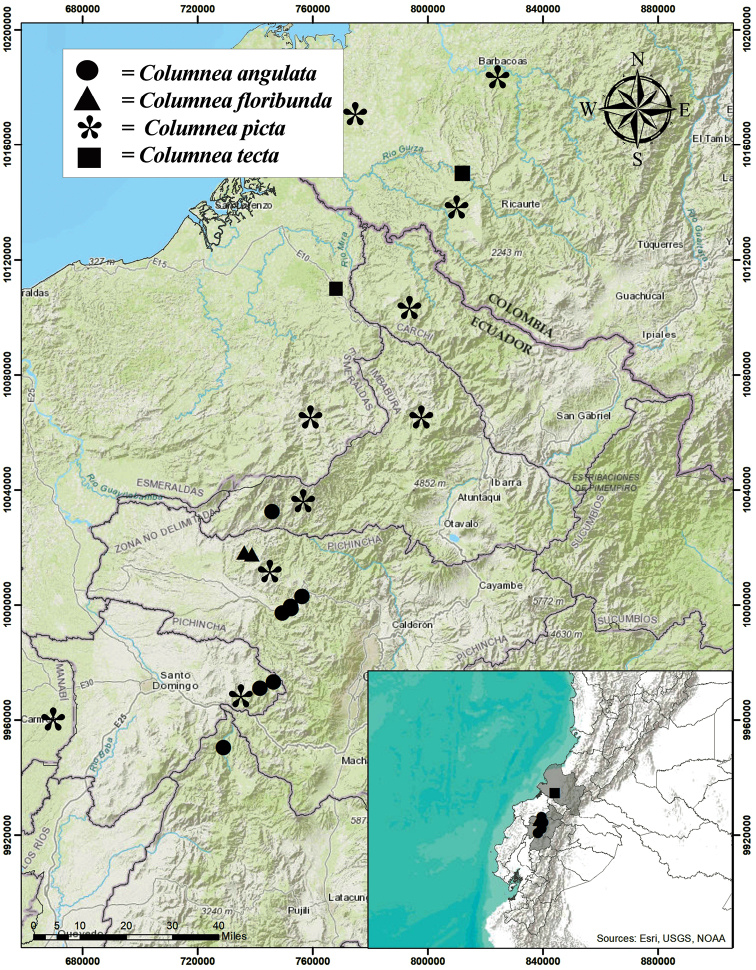
Distribution of *Columneaangulata* (circles), *C.floribunda* (triangles), *C.tecta* (squares), and *C.picta* (asterisks). Note that *C.picta* ranges from southern Ecuador to northwestern Colombia (exceeding the range of the currently described species) (Map generated by Marco Monteros).

The genus *Columnea* is primarily epiphytic. It ranges from Mexico south to Bolivia, and is most diverse in the northern Andes of Colombia and Ecuador. With over 210 species (Clark et al. 2020), *Columnea* is the largest genus in the subfamily Gesnerioideae (Weber et al. 2013, 2020). The genus is distinguished by fruits that are indehiscent berries in contrast to fleshy bivalved capsules in closely related genera. *Columnea* is strongly supported as a monophyletic genus based on molecular phylogenetic studies (Smith et al. 2013; Schulte et al. 2014). The species described here were discovered during exploratory research expeditions and ongoing taxonomic research of herbarium specimens. The three newly described species are similar to many taxa recognized in the section Collandra (Lem.) Benth. or previously classified as members of the genus *Dalbergaria* Tussac. We refrain from classifying the new species to a subgeneric rank because most are artificially defined and not supported by phylogenetic studies (Smith and Carroll 1997; Smith 2000; Clark and Zimmer 2003; Clark et al. 2012; Smith et al. 2013; Schulte et al. 2014). The three species described here are distributed on the western Andean slopes of northern Ecuador and southern Colombia (Fig. 1). Herbarium specimens representing these three species are often annotated as “Columneaaff.picta” or “Columneacf.picta” because they share a similar vegetative feature of apical red leaf apices on the upper and lower leaf surfaces. In contrast, most species of *Columnea* have red leaf apices on the lower leaf surface, but not on the upper leaf surface. Table 1 summarizes prominent characters to differentiate the three new species from each other and from *Columneapicta*.

**Table 1.** General geographic distribution (names in parentheses indicate Ecuadorian provinces and Colombian departments) and comparison of morphological characters between *Columneaangulata*, *C.floribunda*, *C.tecta*, and *C.picta*.

	***Columneaangulata* J.L. Clark & F. Tobar**	***Columneafloribunda* F. Tobar & J.L. Clark**	***Columneapicta* H. Karst**	***Columneatecta* J.L. Clark & L. Clavijo**
Calyx lobe margin	serrate towards apex	serrate	entire	serrate towards apex
Calyx lobe shape	elongate to lanceolate	ovate	broadly ovate	ovate
Corolla posture relative to calyx	oblique to perpendicular	straight	straight	straight
Corolla tube angulation	angulate	not angulate	not angulate	not angulate
Corolla deeply or shallowly bilabiate	shallowly bilabiate (nearly tubular)	shallowly bilabiate (nearly tubular)	deeply bilabiate	shallowly bilabiate (nearly tubular)
Number of flowers/axil	single (rarely 2–3)	4–6	2–3	2–4
Relative length of corolla tube vs. calyx lobes	exceeds length of calyx lobes	exceeds length of calyx lobes	exceeds length of calyx lobes	equal to or less than length of calyx lobes
Distribution	western Andean slopes in northern Ecuador (Cotopaxi, Imbabura, Pichincha, and Santo Domingo de los Tsáchilas)	western Andean slopes of northern Ecuador (Pichincha)	widespread on the western Andean slopes in Colombia & Ecuador	western Andean slopes of northern Ecuador (Esmeraldas) and southern Colombia (Nariño)

## Taxonomic treatment

### Columnea angulata

Taxon classificationPlantaeLamialesGesneriaceae

J.L. Clark & Tobar sp. nov.

B082E489-CCCE-5138-A9C3-54B5E2F9630A

urn:lsid:ipni.org:names:77219739-1

Figs 2 , 3

#### Diagnosis.

Differs from *Columneapicta* by a shallow bilabiate corolla limb (vs. deeply bilabiate corolla limb) and a corolla perpendicular to oblique relative to the calyx (vs. corolla straight relative to the calyx).

#### Type.

Ecuador Imbabura: cantón Cotacachi, parroquia García Moreno, Cordillera de Toisán, Cerro de la Plata, Bosque Protector Los Cedros, sendero Camino del Oso, north of lodge, 0°18'N, 78°46'W, 1500–2600 m, 19 Mar 2003, *J.L. Clark, F. Nicolalde & R. Hall 7413* (holotype: US [US3492386]; isotypes: AAU, COL, K, MO, QCA, QCNE, SEL, UNA).

#### Description.

Facultative epiphyte with dorsiventral shoots to 1.5 m long, subwoody, suffrutescent, glabrescent below, sparsely pilose above; internodes 7–10 cm near base, then clustered at branch apex. Leaves opposite, strongly anisophyllous, papyraceous when dry; larger leaf nearly sessile, petioles 0.1–0.4 cm long, pilose; blade asymmetric, oblanceolate to oblong, 7–28 × 2–9.5 cm, base oblique, apex acuminate, margin serrate, adaxially uniformly dark green to red with dark red apex, glabrous, abaxially light green, upper regions of margins, and apical third of leaf dark red, sparsely pilose to densely pilose along the venation, lateral veins 7–15, primary and secondary veins occasionally red; smaller leaf sessile, sometimes appressed to the dorsal surface of stem; blade asymmetric, lanceolate 0.5–2 × 0.4–0.6 cm, base oblique, apex acuminate, margin serrate, green with red apex on both surfaces, glabrous adaxially, sparsely pilose to densely pilose along the venation and margins abaxially. Inflorescence reduced to a single axillary flower (rarely 2–3); peduncles absent or highly reduced (< 0.2 cm); bracts 1–2, light green, lanceolate, 0.7–1.2 × 0.2–0.4 cm, glabrous on both sides. Flowers subtended by elongate pedicels, 1.5–2.5 cm long, sparsely to densely pilose, tightly appressed to the abaxial leaf surface when immature, becoming pendent during anthesis; calyx lobes 5, nearly free, mostly equal in size and shape, dorsal lobe slightly smaller, lobes appressed to flower when immature and spreading during anthesis, from uniformly yellow, to red with yellow margins, to yellow with a large reddish mid-region, 1.5–3.5 × 0.5–1 cm, ovate to broadly oblong, apex acuminate to acute, margin serrate, pilose on both surfaces; corolla tubular, appearing perpendicular to calyx via a sigmoid-shaped corolla tube, 2.1–3.5 cm long, outer and inner surfaces pilose, base appearing laterally compressed, limb shallowly bilabiate, white suffused with yellow on lower two thirds, more yellow toward apex, splotches of dark red on lower portion of lateral and ventral lobes, light yellow patch below lobes, red streaks abaxially, lobes 0.3–0.4 × 0.3–0.5 cm. Androecium of 4 stamens, filaments connate at the base and forming a filament curtain for 0.2–0.4 cm, free portion of filaments 3–3.5 cm long, glabrous; anthers longer than broad, ca. 2 × 1.5 mm, dehiscing by longitudinal slits; staminode absent; nectary a bilobed dorsal gland, glabrous; ovary superior, densely pilose, 0.2–0.4 × 0.2 cm, style ca. 2.5 cm long, glabrous, stigma included and shallowly bifid. Fruit not observed.

#### Phenology.

This species has been found with flowers in two periods: February to May and August to October.

#### Etymology.

The specific epithet is in reference to the angulate or bent corolla tube. The corolla is nearly perpendicular to the calyx lobes because of the sigmoid-shaped tube.

#### Distribution and preliminary assessment of conservation status.

*Columneaangulata* is locally abundant in forests along the western slopes of the Ecuadorian Andes in the provinces of Cotopaxi, Imbabura, Pichincha, and Santo Domingo de los Tsáchilas (Fig. 1) where it grows in mature forests and the shaded understory of recently cleared forests, from 1500 to 2600 m in elevation. It is especially common along the old highway between Quito and Santo Domingo. It has been documented in two protected areas: Reserva Florística-Ecológica Río Guajalito and Bosque Protector Los Cedros. According to the IUCN Red List criteria (IUCN 2001) for limited geographic range (B1, less than 20,000 km^2^) and considering the uncertain future of habitat conservation of western Andean forests (B2b, c), *Columneaangulata* should be listed in the category Vulnerable (VU).

#### Comments.

**Figure 2.**
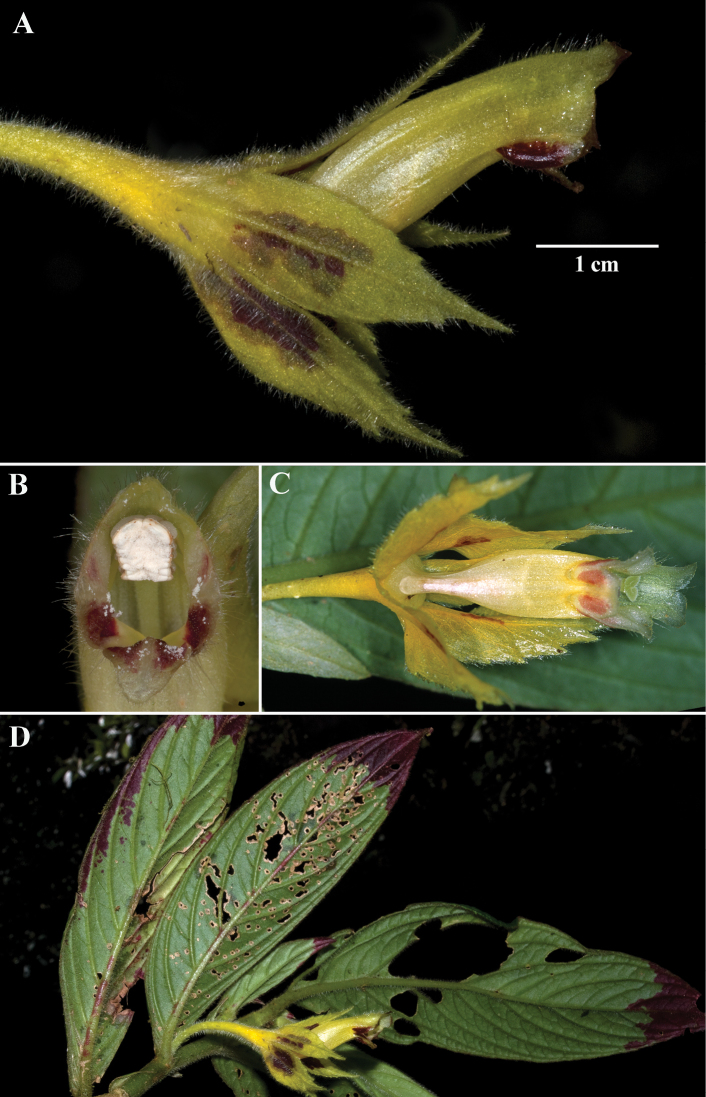
*Columneaangulata* J.L. Clark & F. Tobar **A** mature flower **B** front view of flower **C** ventral view of flower showing laterally compressed corolla tube **D** dorsiventral habit (**A, D** from *J.L. Clark et al. 12198***B** from *J.L. Clark 10968***C** from *J.L. Clark et al. 7413*). Photos by J.L. Clark.

**Figure 3.**
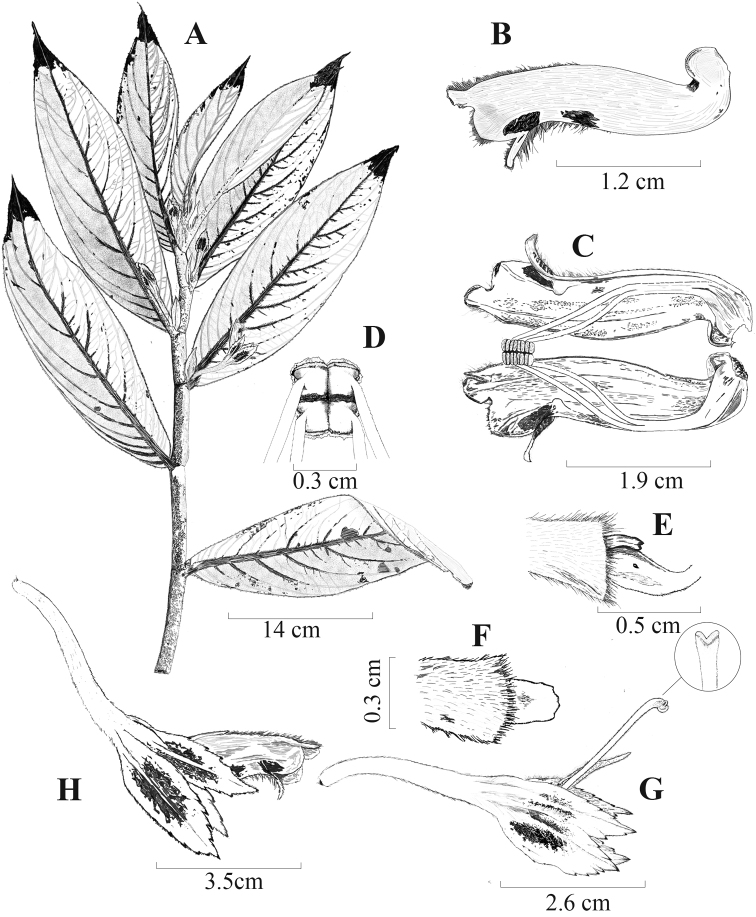
*Columneaangulata* J.L. Clark & F. Tobar **A** dorsiventral habit **B** lateral view of flower **C** dissected corolla showing filaments and ventral view of the anthers **D** dorsal view of the anthers **E** lateral view of ovary and nectary gland **F** dorsal view of nectary gland **G** lateral view of calyx and style, showing shallowly bifid stigma **H** lateral view of flower. Illustrated by M.J. Gavilanes, based on *F. Tobar et al 3409*.

*Columneaangulata* is unique from other *Columnea* by the posture of the pendent mature flowers where the corolla tubes are oriented oblique to perpendicular relative to the calyx (Figs 2A, 3B). Another defining character is a constriction at the base of the corolla tube that makes it appear laterally compressed (Fig. 2C). *Columneapicta* and *C.angulata* are vegetatively similar and grow sympatrically. These two species are differentiated by the presence of deeply bilabiate corolla tubes in *Columneapicta* (Fig. 6A) in contrast to the shallowly bilabiate corolla tubes in *C.angulata* (Fig. 2B, C). *Columneaangulata* differs from *C.tecta* by an elongate corolla tube (vs. corolla tube that does not exceed the calyx lobes in *C.tecta*) and single axially flowers (rarely 2–3) in contrast to the abundant clusters of 3–5 axially flowers in *C.floribunda*. *Columneapicta* and *C.angulata* are the two most commonly collected species in this complex and readily differentiated by the entire calyx margin in *C.picta* and serrate calyx margin in *C.angulata*.

#### Specimens examined.

Ecuador Cotopaxi: cantón Sigchos, parroquia San Francisco de las Pampas, Bosque Integral Otonga, 0°25.17'S, 79°0.19'W, 1900 m, 26 Jan 2001, *J.L. Clark and Muñoz 6125* (QCA, QCNE, SEL, UNA, US); Pichincha: cantón Quito, parroquia Nono, El Pahuma Orchid Reserve, 17 km east of Nanegalito, 0°1'S, 78°37'W, 1700 m, 17 Apr 2003, *J.L. Clark et al. 7648* (QCA, QCNE, SEL, UNA, US); cantón San Miguel de los Bancos, Mindo Loma Cloud Forest Reserve, km 73.5 via Calacali-La Independencia, 3 km past the entrance to the village of Mindo, 0°0'44"S, 78°44'29"W, 1800 m, 23 May 2011, *J.L. Clark & C. Aulestia 12198* (QCNE, UNA, US); cantón San Miguel de los Bancos, Las Gralarias Reserve, 1.2 km east of the lodge, 0°05'N, 78°43'W, 1900 m, 15 Aug 2017, *F. Tobar, A. Nieto, A. Marcayata & S. Imba 2832* (QCA); cantón San Miguel de los Bancos, Las Gralarias Reserve, Puma trail, 0°05'N, 78°43'W, 1900 m, 21 May 2018, *F. Tobar, F. Richter 3280* (QCA); cantón San Miguel de los Bancos, Puyucunapi Reserve, cultivada cerca de la casa de la reserva, 0°01'N, 78°41'W, 1800 m, 13 Oct 2019, *F. Tobar & M. Gavilanes 3409* (HPUCESI, QCNE); cantón San Miguel de los Bancos, a 2.6 km al este de San Tadeo en la vía a Bellavista Lodge, 0°01'N, 78°44'W, 1893 m, 11 Mar 2020, *F. Tobar & M. Gavilanes 3475* (QCNE); cantón San Miguel de los Bancos, Puyucunapi Reserve, a 800 m de la entrada del transecto principal, 0°01'N, 78°41'W, 1995 m, 12 Mar 2020, *F. Tobar & M. Gavilanes 3479* (QCA); **Santo Domingo de los Tsáchilas**: cantón Santo Domingo de los Colorados, Bosque Protector Rio Guajalito, located on the old Quito-Santo Domingo road, between Chiriboga and La Palma, 0°18'50"S, 78°55'35"W, 1796 m, 30 May 2009, *J.L. Clark et al. 10968* (NY, QCNE, SEL, US); Reserva Florística-Ecológica Río Guajalito, km 59 de la carretera antigua Quito-Sto. Domingo de los Colorados, a 3.5 km al NE de la carretera, 0°13'53"S, 78°48'10"W, 1800–2200 m, 3 Apr 2003, *J.L. Clark, N. Muchhala & A. Hoyos 7618* (QCA, QCNE, SEL, UNA, US).

### Columnea floribunda

Taxon classificationPlantaeLamialesGesneriaceae

Tobar & J.L. Clark sp. nov.

A0BDCEBC-3855-5347-8B0F-8C6C98451795

urn:lsid:ipni.org:names:77219740-1

Figs 4 , 5

#### Diagnosis.

Differs from *Columneapicta* by a nearly tubular corolla (vs. deeply bilabiate corolla). Differs from *Columneaangulata* by the straight corolla relative to the calyx (vs. oblique to perpendicular corolla relative to the calyx). Differs from *Columneatecta* by corollas that exceed the length of the calyx lobes (vs. corollas that are equal to or less than the length of the calyx lobes).

#### Type.

Ecuador Pichincha: cantón Pichincha, parroquia Pacto, Bosque Protector Mashpi, sendero Mashpi Capuchin, 5 km al norte de Lodge, 0°09'N, 78°52'W, 900–1200 m, 18 Jan 2020, *F. Tobar, C.H. Graham, T. Santander & E. Guevara 3527* (holotype: QCA; isotypes: QCNE, US).

#### Description.

Facultative epiphyte with dorsiventral shoots to 2–3 m long, subwoody, suffrutescent, glabrescent below, sparsely pilose above; internodes 3–16 cm near base, then clustered at branch apex. Leaves opposite, strongly anisophyllous, papyraceous when dry; larger leaf nearly sessile, petioles 0.1–0.2 cm long, pilose; blade asymmetric, oblanceolate to oblong, 1–28 × 4.8–6.2 cm, base oblique, apex acuminate, margin serrate, adaxially uniformly dark green with bright red, glabrous, abaxially light green with bright red apex, sparsely pilose, lateral veins 7–12; smaller leaf sessile, blade asymmetric, lanceolate 1.4–2.5 × 0.3–0.5 cm, base oblique, apex acuminate, margin serrate, green with red apex on both surfaces, adaxially glabrous, abaxially sparsely pilose. Inflorescence reduced to axillary clusters of 3–5 flowers; peduncles absent or highly reduced (< 0.2 cm long); bracts 1–2, light green, oblong, 0.5–1.2 × 0.2–0.3 cm, glabrous on both sides. Flowers subtended by elongate pedicels, 2.2–3.3 cm long, sparsely pilose; calyx lobes 5, nearly free, mostly equal in size and shape, dorsal lobe elongate and slender, 1.7–2.1 × 0.7–1.3 cm, ovate, apex acute, margin serrate, mostly yellow with red splotches in the center, inner and outer surfaces pilose; corolla tubular, 0.6–2.1 cm long, mostly yellow with whitish base, outer and inner surfaces pilose, limb shallowly bilabiate, corolla lobes 0.3–0.5 × 0.2–0.4 cm, lateral and lower lobes red, upper lobes yellow. Androecium of 4 stamens, filaments connate at the base and forming a filament curtain for 0.2–0.3 cm, free portion of filaments 1.5–1.9 cm long, minutely pubescent; anthers longer than broad, ca. 0.3 × 0.2 mm, dehiscing by longitudinal slits; staminode absent; nectary a trilobed dorsal gland, glabrous; ovary superior, densely pilose, 0.2–0.4 × 0.2 cm, style 1.5–1.8 cm long, minutely pubescent, stigma included and shallowly bifid. Fruit an indehiscent globose white berry. Seeds not observed.

#### Phenology.

Collections of *Columneafloribunda* are documented with flowers between January and April and between June and October. Fruits have been recorded during March.

#### Etymology.

The specific epithet refers to axillary clusters of several flowers (3–5).

#### Distribution and preliminary assessment of conservation status.

*Columneafloribunda* is locally abundant in the Mashpi Rainforest Biodiversity Reserve (900–1340 m) and the surrounding roads, a Chocó biogeographic forest relict in northern Ecuador. It is likely that additional populations are located in the adjacent provinces of Imbabura and Esmeraldas. Future research expeditions to unexplored areas of the Cotacachi Cayapas Ecological Reserve will hopefully result in additional documented populations of *C.floribunda*. According to the IUCN Red List criteria (IUCN 2001) for limited geographic range (B2a, less than five locations) and considering the uncertain future of habitat conservation, *Columneafloribunda* should be listed in the category Endangered (EN).

#### Comments.

**Figure 4.**
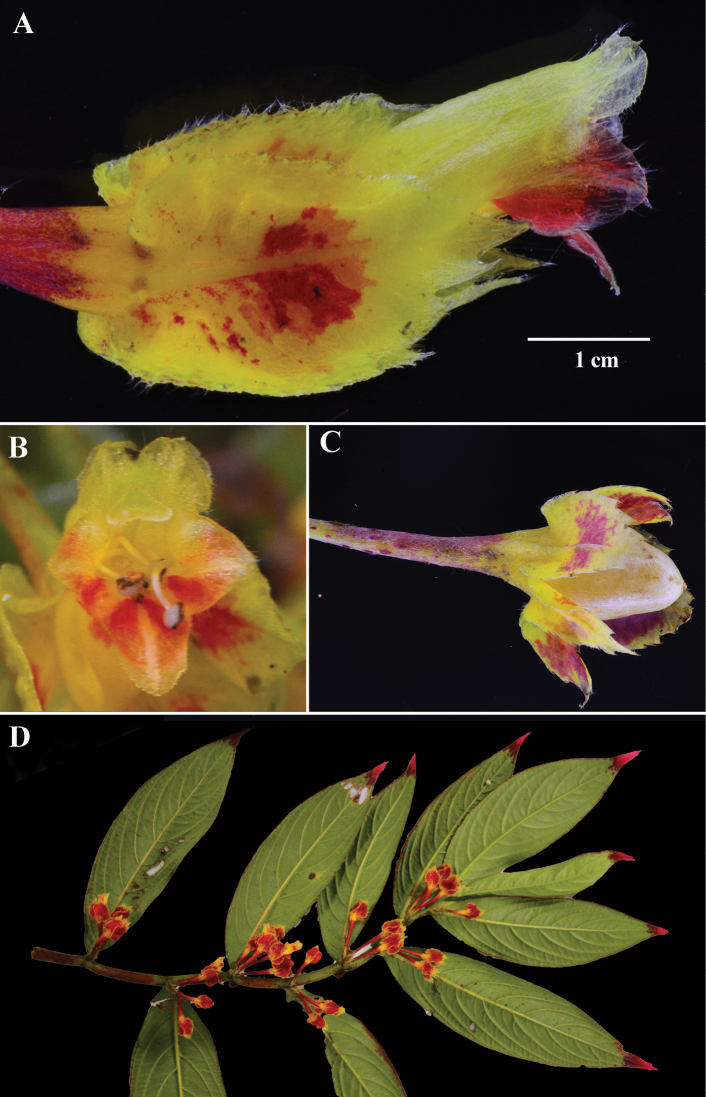
*Columneafloribunda* F. Tobar & J.L. Clark **A** mature flower **B** front view of flower during anthesis **C** mature fruit **D** dorsiventral habit **A–D** from *F. Tobar et al. 3527* (**A–D** from *F. Tobar et al. 3527*, holotype). Photos by F. Tobar.

*Columneafloribunda* is readily distinguished from all other congeners by the elongate corolla tubes that exceed the length of the calyx lobes (Fig. 4A), in contrast to the corolla tubes of *C.tecta* that are equal to or less than the length of the calyx lobes (Fig. 7C); the corolla posture relative to the calyx that is straight (Fig. 4A), in contrast to the oblique to perpendicular corolla relative to the calyx of *C.angulata* (Fig. 2A); and the axillary clusters of three or more flowers (Fig. 4D).

**Figure 5.**
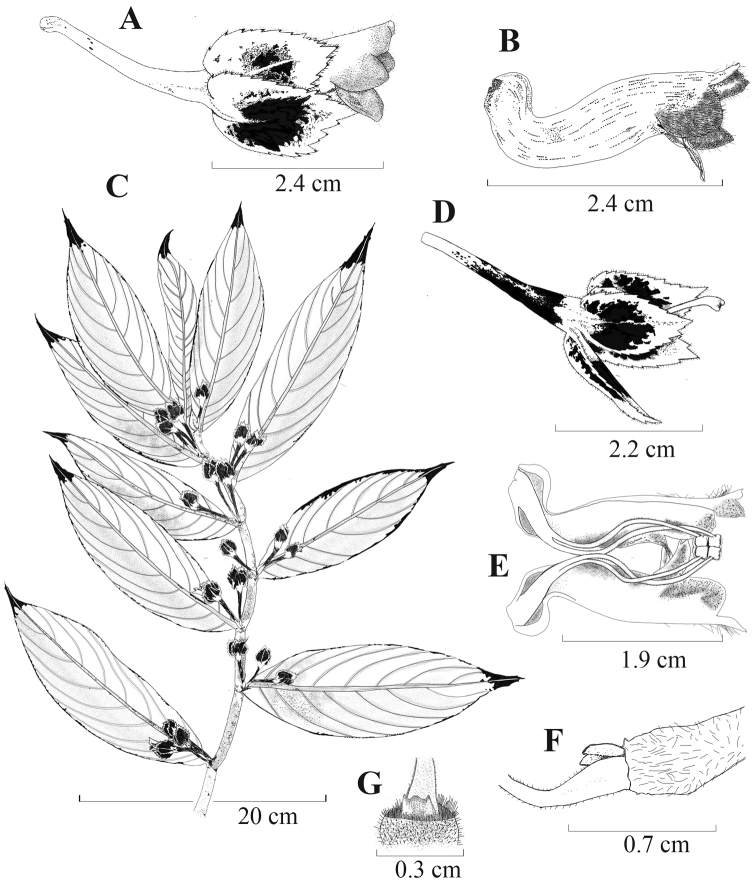
*Columneafloribunda* F. Tobar & J.L. Clark **A** mature flower **B** Lateral view of corolla **C** dorsiventral habit **D** lateral view of calyx and style, showing shallowly bifid stigma **E** corolla dissected showing filaments and ventral view of anthers **F** lateral view of ovary and nectary gland **G** dorsal view of nectary gland. Illustrated by M.J. Gavilanes, based on *F. Tobar et al. 3527*, holotype.

#### Specimens examined.

Ecuador Pichincha: cantón Pacto, Mashpi Lodge, transecto Mashpi Laguna, a 500 m de la entrada del transecto, 0°09'N, 78°52'W, 880 m, 21 Sep 2017, *F. Tobar & A. Nieto 2903* (QCA); cantón Pacto, transecto Mashpi Capuchin, entrada del transecto, 0°10'N, 78°52'W, 800 m, 20 Mar 2018, *F. Tobar, A. Marcayata & K. Cortez 3161* (QCA); cantón Pacto, km 20, carretero entre La Delicia y el pueblo de Mashpi, 0°09'N, 78°51'W, 1200 m, 18 Dec 2019, *F. Tobar & M. Gavilanes 3509* (QCA); cantón Pichincha, Amagusa Reserve, 1200 m dentro del sendero principal hacia el rio, 0°09'N, 78°51'W, 1213 m, 18 Mar 2018, *F. Tobar, C. Poveda, S. Basantes & M. Gavilanes* 3465 (HPUCESI, QCNE); cantón Pacto, Mashpi reserve, road to lodge, 0°09'38"N, 78°50'58"W, 1338 m, 7 Feb 2019, *M. Perret & F. Tobar 258* (QCNE).

### Columnea tecta

Taxon classificationPlantaeLamialesGesneriaceae

J.L. Clark & Clavijo sp. nov.

85FF799B-EF65-5EB0-8CCB-110C1D62AA52

urn:lsid:ipni.org:names:77219741-1

Fig. 7

#### Diagnosis.

Differs from *Columneapicta* by a nearly tubular corolla (vs. deeply bilabiate corolla) that is equal to or shorter than the calyx lobes (vs. corolla that extends beyond the calyx lobes).

#### Type.

Ecuador Esmeraldas: cantón San Lorenzo, remnant patch of forest along highway Ibarra-San Lorenzo, between the towns of Durango and Alto Tambo, 0°57'21"N, 78°33'38"W, 664 m, 3 Jun 2009, *J.L. Clark & 2009 Gesneriad Research Expedition Participants 11104* (holotype: US [3693986]; isotypes: MO, NY, QCNE, SEL).

#### Description.

Facultative epiphyte with dorsiventral shoots to 1.5 m long, subwoody, suffrutescent, glabrescent below, sparsely pilose above; internodes 5–10 cm near base, then clustered at branch apex. Leaves opposite, strongly anisophyllous, papyraceous when dry; larger leaf nearly sessile, petioles succulent, 0.3–0.8 cm long, glabrous; blade asymmetric, broadly oblanceolate, 7–30 × 3–6.6 cm, base oblique, apex acuminate, margin serrate, adaxially uniformly green with bright red apex, glabrous, abaxially light green with bright red apex, sparsely pilose along the venation, lateral veins 7–14, primary vein bright red, secondary veins red at base and green adaxially; smaller leaf sessile and often clasping the base of the stem; blade asymmetric, lanceolate 0.5–1.5 × 0.4–0.5 cm, base oblique, apex acuminate, margin serrate, green with red apex on both sides, adaxially glabrous, abaxially sparsely pilose. Inflorescence reduced, appearing in clusters of 1–4 axially flowers; peduncles absent or highly reduced (< 0.2 cm long); bracts 1–2, light green, oblanceolate, 0.7–1 × 0.2–0.3 cm, glabrous on both sides. Flowers subtended by elongate pedicels, 1.5–2.4 cm long, sparsely pilose, with enations near the apex; calyx lobes 5, nearly free, mostly equal in size and shape, dorsal lobe slightly smaller, 1.5–2.3 × 1–2 cm, ovate, apex acute, margin serrate, yellow with red splotches in the center, inner and outer surfaces sparsely pilose; corolla tubular and erect, 1–1.9 cm long, outer and inner surfaces pilose, limb shallowly bilabiate, mostly yellow with red striations on lateral and ventral lobes, corolla lobes 0.2–0.5 × 0.2–0.4 cm. Androecium of 4 stamens, filaments connate at the base for 0.1–0.3 cm and forming a filament curtain, free portion of filaments ca. 1.5 cm long, glabrous; anthers longer than broad, ca. 2 × 1.5 mm, dehiscing by longitudinal slits; staminode absent; nectary a bilobed dorsal gland, glabrous; ovary superior, densely pilose, 0.2–0.4 × 0.2 cm, style ca. 1.4 cm long, glabrous, stigma included and shallowly bifid. Fruit an indehiscent oblong white berry, 1.2 × 0.72 cm.

#### Phenology.

This species was documented with flowers in June and May. Fruits have been recorded in June.

#### Etymology.

The specific epithet tecta refers to the “hidden” or relatively short corolla tube that does not exceed the length of the calyx lobes, an unusual character in *Columnea*.

#### Distribution and preliminary assessment of conservation status.

This species has not been found in any formally protected areas. According to the IUCN Red List criteria (IUCN 2001) for limited geographic range (B2a, less than five locations) and considering the uncertain future of habitat conservation of western Andean forests, *Columneatecta* should be listed in the category Endangered (EN).

#### Comments.

**Figure 6.**
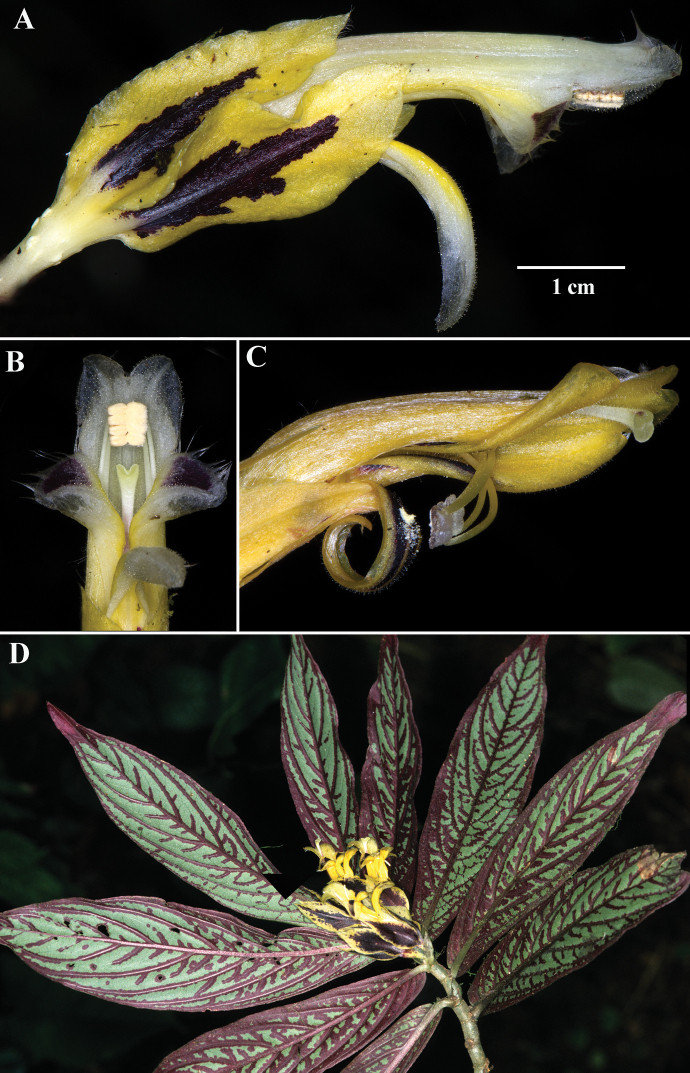
*Columneapicta* H. Karst **A** lateral view of flower featuring deeply bilobed corolla **B** ventral view of flower during anthesis **C** mature flower featuring curled lower lobe **D** dorsiventral habit (**A, B** from *J.L. Clark & L. Jost 16301***C** from *J.L. Clark et al. 15393***D** from *J.L. Clark, M. Mailloux & S. Seger 7942*). Photos by J.L. Clark.

**Figure 7.**
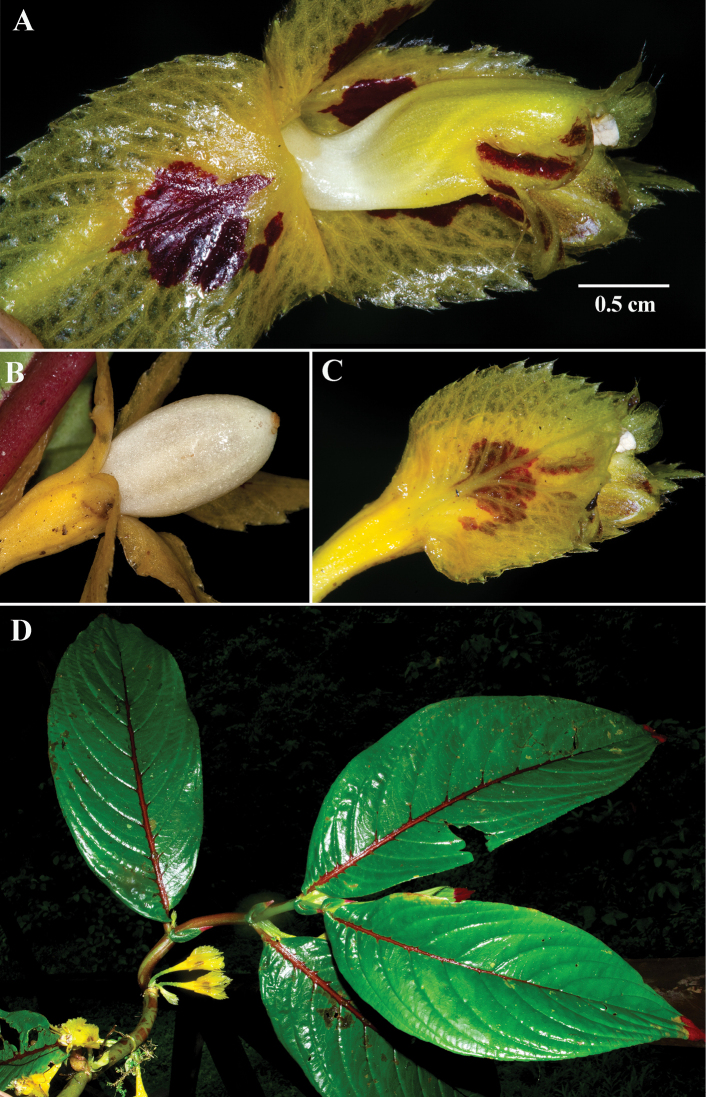
*Columneatecta* J.L. Clark & Clavijo **A** mature flower with lateral calyx lobe pulled back **B** Oblong white berry **C** mature flower **D** dorsiventral habit (**A–D** from *J.L. Clark et al. 13433*). Photos by J.L. Clark.

*Columneatecta* is readily distinguished from all other congeners by relatively short corollas that barely exceed the length of the calyx lobes (Fig. 7). The corollas of *Columneatecta* have limbs that are shallowly bilabiate (Fig. 7A) in contrast to the deeply bilabiate corollas of *Columneapicta* (Fig. 6A). *Columneatecta* and *C.picta* are vegetatively similar by the presence of a dorsiventral habit with red apices on both leaf surfaces. The corolla tubes of *C.tecta* are short (less than the length of the calyx lobes and shallowly bilabiate) relative to the longer corolla tubes of *C.picta* (exceeding the length of the calyx lobes and deeply bilabiate). *Columneatecta* differs from *C.angulata* by a straight orientation of the corolla relative to the calyx (vs. oblique to perpendicular in *C.angulata*).

#### Specimens examined.

Colombia Nariño: municipio Barbacoas, corregimiento El Diviso, western slopes of the Cordillera Occidental, trail from El Diviso towards Río Güiza, 1°21'21"N, 78°11'45"W, 404 m, 13 May 2013, *J.L. Clark, L. Clavijo, O. Marín & M. Flores 13433* (COL, CUVC); Altaquer to Junín, near Altaquer, 10 May 1972, *H. Wiehler, R.L. Dressler, N.H. Williams & N.F. Williams 72222* (SEL).

## Supplementary Material

XML Treatment for Columnea angulata

XML Treatment for Columnea floribunda

XML Treatment for Columnea tecta
